# Optical Properties of Site-Selectively Grown InAs/InP Quantum Dots with Predefined Positioning by Block Copolymer Lithography

**DOI:** 10.3390/ma14020391

**Published:** 2021-01-14

**Authors:** Paweł Holewa, Jakub Jasiński, Artem Shikin, Elizaveta Lebedkina, Aleksander Maryński, Marcin Syperek, Elizaveta Semenova

**Affiliations:** 1 Laboratory for Optical Spectroscopy of Nanostructures, Faculty of Fundamental Problems of Technology, Wrocław University of Science and Technology, Wyb. Wyspiańskiego 27, 50-370 Wrocław, Poland; jakub.jasinski@pwr.edu.pl (J.J.); olek.marynski@gmail.com (A.M.); 2 DTU Fotonik, Technical University of Denmark, DK-2800 Kgs. Lyngby, Denmark; artem.shikin@phystech.edu (A.S.); lile@fotonik.dtu.dk (E.L.)

**Keywords:** site-selective growth, quantum dots, photoluminescence

## Abstract

The InAs/InP quantum dots (QDs) are investigated by time-integrated (PL) and time-resolved photoluminescence (TRPL) experiments. The QDs are fabricated site-selectively by droplet epitaxy technique using block copolymer lithography. The estimated QDs surface density is ∼1.5 × 10^10^ cm^−2^. The PL emission at T=300 K is centered at 1.5 μm. Below T=250 K, the PL spectrum shows a fine structure consisting of emission modes attributed to the multimodal QDs size distribution. Temperature-dependent PL reveals negligible carrier transfer among QDs, suggesting good carrier confinement confirmed by theoretical calculations and the TRPL experiment. The PL intensity quench and related energies imply the presence of carrier losses among InP barrier states before carrier capture by QD states. The TRPL experiment highlighted the role of the carrier reservoir in InP. The elongation of PL rise time with temperature imply inefficient carrier capture from the reservoir to QDs. The TRPL experiment at T=15 K reveals the existence of two PL decay components with strong dispersion across the emission spectrum. The decay times dispersion is attributed to different electron-hole confinement regimes for the studied QDs within their broad distribution affected by the size and chemical content inhomogeneities.

## 1. Introduction

Self-assembled semiconductor InAs quantum dots (QDs) grown on a standard InP(100) substrate are prospective candidates for an active part of optoelectronic devices. The InAs/InP QD material composition in line with the quasi-0D density of states [1,2] and relatively strong carrier confinement [1,2,3] make these QDs attractive for lasers and optical amplifiers operating in the near-infrared spectral range above 1.5 μm, important for telecom applications [4,5,6,7]. Despite the successful realization of InAs/InP QDs-based lasers and optical amplifiers [4,5,8,9,10], their parameters are still far from expected [11,12]. The cause may be found in carrier leakage from QDs, lowering the modal gain, increasing threshold current density, lowering the energy conversion efficiency, and introducing the temperature instability of device parameters. Another detrimental factor is QDs size uniformity, negatively imprinting optical gain inhomogeneity. Finally, poor control over the QDs surface distribution influences repeatability in a device fabrication process. Most of these problems come directly from the QDs fabrication method.

The most widely used Stranski–Krastanov (SK) QD growth technique relies on the roughly 3.2% lattice mismatch between InAs and InP materials. During the growth process, first, a thin, highly-strained InAs quantum well (QW) is formed, called wetting layer (WL), and next, nucleation of QDs occurs in a self-assembled way following the strain relaxation process [13]. Despite the critical role of the WL serving as the carrier reservoir for QDs [14], at elevated temperatures typical for device operation, the WL is perceived as an efficient carrier escape channel from QDs, negatively affecting device parameters [15] as stated in the previous paragraph. Moreover, the self-assembly process of SK QDs results in poor control over the QD size distribution leading to its substantial inhomogeneous broadening. On the one hand, the substantial inhomogeneous broadening can be highly beneficial for broad-band QD-based optical amplifiers. On the other hand, it is detrimental to the optical gain in the laser resonator due to the limited number of QDs participating in the lasing process.

A few proposed solutions can mitigate the carrier leakage problem in the SK QDs, including dot-in-a-well approach [16], graded-index separate-confinement-heterostructure [17], stacking many QD layers [18], applying the tunnel-injection scheme [19], or fabricating QDs without the WL. For the latter, one can use the droplet epitaxy (DE) technique in which a group-III element droplet is deposited directly on a substrate undergoing subsequent re-crystallization under deposition of a group-V ad-atoms, spontaneously forming a nanometer-sized island [20]. First applied to the formation of GaAs QDs on ZnSe substrate [21], later successfully employed to other semiconductor systems, including the InAs/InP one [22]. However, the DE is still a self-assembled process for which the control over the QD size, shape, and surface density is limited. This issue can be addressed by the selection of QD nucleation sites.

Several techniques have been used for site selection in InP material, including local atomic-force-microscopy oxidation [23] or nanopatterning by electronic lithography [24]. Block copolymer lithography was previously used to form a mask for selective-area growth (SAG) of In(Ga)As QDs in both GaAs [25,26,27] and InP [13,25,26,28,29] material systems. This method can result in establishing a dense ensemble of InAs/InP QDs [13,26,28,29,30]. Within this growth approach, the nucleation of QDs occurs in openings of a few tens of nanometers in size. Thus, the control over the mask preparation [31,32,33,34] and the semiconductor material deposition process [35] allows for the fabrication of uniform nanopattern with QDs and the control over their optical properties to a great extent as compared to the standard SK growth technique [13,26].

This work shows the optical properties of site-selective droplet epitaxy (DE) InAs/InP QDs fabricated in a novel approach using di-block copolymer lithography in metalorganic vapor-phase epitaxy (MOVPE). After initial patterning of an InP substrate with nanopits array, the mask is completely removed. Therefore, QDs nucleation from indium droplets under arsine atmosphere occurs not in mask openings but directly in nanopits etched in situ in MOVPE on the InP substrate. The resultant QDs have a multimodal distribution. This allows for the comprehensive studies on mutual dependencies between QD families in their dense ensemble, including charge transfer between the dots within an individual mode and between different modes, properties of carrier confinement for the dots of different sizes concentrated in a well-defined QDs family, and carrier capture and re-excitation for an individual QD family controlled by the temperature via the variation of the phonon population. The optical research is conducted using the time-integrated and time-resolved photoluminescence (PL and TRPL) experiments as a function of excitation power density ($0.05\;W/cm^{2}<P_{\mathrm{exc}}<30\;W/cm^{2}$) and temperature (15 K<T<300 K). The gathered knowledge is a step towards understanding the carrier confinement properties of the site-selective DE InAs/InP QDs grown using a predefined mask prepared by the di-block copolymer lithography.

## 2. Results and Discussion

### 2.1. Investigated Structure

**Figure 1:**
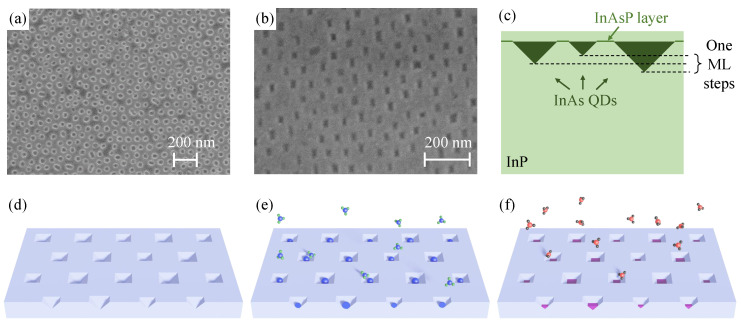
(**a**,**b**) SEM images of (**a**) hard silicon oxycarbide mask formed after oxygen plasma treatment in ICP-RIE, (**b**) the InP surface after CBr${}_{4}$ etching in the MOVPE reactor and the subsequent oxycarbide mask removal. (**c**) The scheme of the InAs QDs ensemble embedded in the InP substrate. (**d**–**f**) A simplified scheme of the QDs deposition process: (**d**) nanopits in InP, (**e**) indium deposition and formation of indium droplets in nanopits, (**f**) re-crystalization of QDs under the arsine atmosphere.

The fabrication of the investigated structure is split into three stages: (a) fabrication of the oxycarbide mask, (b) fabrication of nanopits in InP, and (c) deposition of QDs. Each of these steps is explained in detail in the *Experimental and Theoretical Methods* section (Section 3). Here we present the summary of the fabrication procedure. The mask is fabricated out of the polystyrene-block-polydimethylsiloxane (PS-*b*-PDMS) copolymer spin-casted onto InP. The solvent annealing promotes the formation of the standing PS cylinder pattern in the PDMS matrix. Subsequently, in the dry etching process, the copolymer matrix is turned into the oxycarbide mask with voids in the places of PS cylinders. Figure 1a presents the scanning electron microscope (SEM) image of the silicon oxycarbide mask. The nanopits are then etched in InP in the MOVPE reactor with carbon tetrabromide (CBr${}_{4}$) and the mask is removed by hydrofluoric acid (HF). After the mask removal, the nanopits in InP are inspected by the SEM (Figure 1b). These openings have faceting by {111} family planes with the elongation due to the etching anisotropy between {111}A and {111}B InP planes [35], resulting in a triangle-like cross-section of a nanopit (Figure 1c). The subsequent QDs nucleation process is schematically presented in Figure 1d–f: void nanopits of slightly different sizes (Figure 1d) are exposed to the flow of trimethylindium (TMIn) particles, and indium droplets (blue spheres) are formed in the nanopits (Figure 1e). Then, under arsine (AsH${}_{3}$) flow, As atoms (red spheres) turn In droplets into InAs QDs (purple pyramids) (Figure 1f). Finally, QDs are capped by the InP layer and annealed in phosphine ambient. Note that the annealing in the arsine ambient promotes the As-P exchange on the InP surface, leading to the formation of a thin, inhomogeneous (thickness and chemical content variation) InAsP layer, and subsequently the InP/InAsP/InP QW after the InP deposition, which resembles a WL for the SK QDs.

### 2.2. Excitation-Power-Dependent PL Experiment

**Figure 2:**
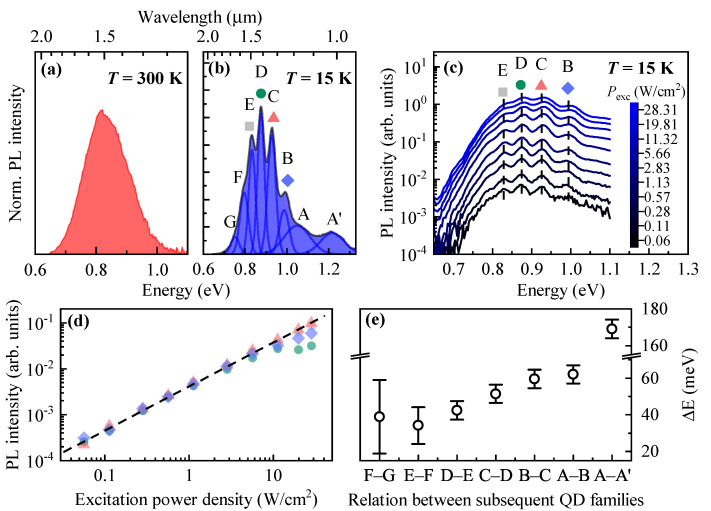
(**a**) Room-temperature PL spectrum for site-selective DE InAs/InP QDs (red shaded area). (**b**) The PL spectrum (blue shaded area) at T=15 K revealing the fine structure indicating a multimodal distribution of QDs. Capital letters A-G and symbols (if applied) label subsequent QDs distributions/PL modes. The letter A’ indicates 0D-like shallow confined states possibly related to the existence of an InAsP QW on top of the QD array. The solid black lines represent the multiple Gaussian-line shapes fitting the PL spectrum. (**c**) Excitation power density-dependent evolution of the PL spectrum plotted in a semi-log scale. (**d**) The PL intensity of selected modes B, C, D, and E [symbols as in (**c**)] as a function of excitation power density. (**e**) The energy distance between subsequent PL modes (QD families).

The room-temperature PL spectrum of site-selective DE InAs/InP QDs is presented in Figure 2a. The PL is centered at the telecom-relevant spectral window near 1.55 μm (∼0.8 eV) and shows a significant broadening of nearly 180 meV related to, e. g., the QDs size distribution, carrier-phonon coupling, and carrier redistribution processes. Good visibility of PL emission at moderate excitation power density ($P_{\mathrm{exc}}\approx 1\;W/cm^{2}$) reflects good carrier confinement in the dots as predicted by relatively large band offsets in the conduction (∼320 meV) and valence (∼530 meV) [36] bands for strained InP and InAs materials. Moreover, it may suggest a relatively low density of non-radiative recombination centers in the vicinity of QDs. Interestingly, at T=300 K, there is no evidence for a multimodal QDs distribution in the emission spectrum. It can be resolved by decreasing the temperature and it is fully manifested at cryogenic temperatures.

Figure 2b shows the PL spectrum from QDs at T=15 K. The emission pattern reveals the fine structure consisting of a series of individual PL bands labeled with letters from A’ to G. This spectrum is fitted with a Gaussian-line shape profiles (see Figure 2b, solid blue lines) to extract their mean energy position and the peak area equivalent to the integrated PL intensity. The excitation power density-dependent PL experiment, displayed in Figure 2c, reveals the nature of individual PL bands. The attention is paid to PL bands B to E for which the fitting procedure gives the most reliable results. Figure 2d shows the extracted PL intensities (symbols as in Figure 2c) as a function of $P_{\mathrm{exc}}$ plotted in the log-log scale. The PL scales for all the PL bands in the same way with $P_{\mathrm{exc}}$ over almost three decades. This observation excludes the interpretation in which the individual PL modes originate from the emission of confined higher QD states [1]. Therefore, one can assume that PL in each band is mainly governed by the recombination of electron-hole (e-h) pairs confined in the QD ground states. Although the structure is nominally undoped, some residual *p*-type doping may be present, resulting in the recombination of charged e-h complexes, which should not significantly imprint the overall interpretation of the presented data.

The above-stated assumption on recombination processes, justified by the excitation power-dependent PL experiment, is essential for the analysis of individual PL peak energy positions. Since each of the individual PL bands is contributed by emission from the size-distributed QDs, and the QD ground state emission energy (*E*) is governed mainly by the dot height (*H*), the energy difference between subsequent PL bands should scale in a non-linear way according to $E\propto 1/H^{2}$. Figure 2e shows that this scenario can be realized for the studied QDs. The energy distance between subsequent PL bands monotonically decreases in the range of 40–60 meV with decreasing the PL peak energy, and so the dot height in the individual PL modes A to G as well. The observation of multimodal emission in site-selective DE QDs is consistent with that for SK InAs/InP QDs [1,37,38,39,40,41,42,43,44] and SK InAs/GaAs QDs [45]. However, the reason for the formation of multimodal QDs presented in this work can be different due to the involved fabrication procedure.

**Figure 3:**
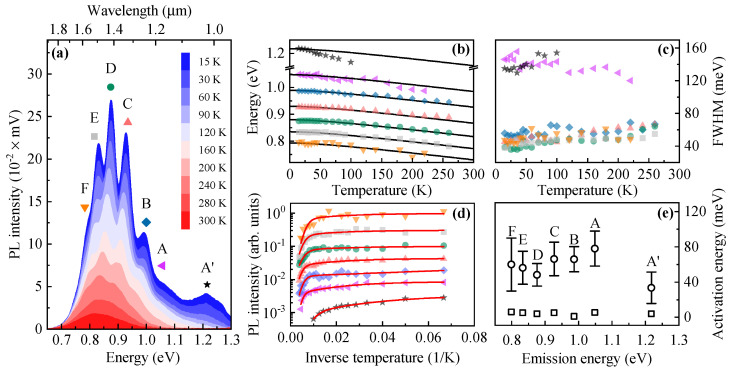
(**a**) Temperature-driven evolution of the PL spectrum from multimodal site-selective DE InAs/InP QDs. Capital letters and symbols (if applied) label subsequent PL modes. (**b**) Temperature dependence of the PL peaks energy. Solid black lines show temperature-driven bandgap changes for InAs according to the Varshni formula (Equation (1), curves are shifted in energy). (**c**) The FWHM (peak width) vs. temperature [symbols as in (**a**)]. (**d**) Temperature-driven PL intensity quench [symbols as in (**a**)]. Solid red lines are fitted to the data accordingly to Equation (2). (**e**) The extracted PL intensity quench energies $E_{1}$ (squares) and $E_{2}$ (circles) from (**d**) as a function of the PL mode emission energy.

Besides the major role of the QD height, the QD emission energy is influenced also by other parameters. Figure 2b shows that individual PL modes are characterized by significant spectral broadening approximated by the Full-Width-at-Half-Maximum (FWHM) of the Gaussian profiles. For QD families B to E, the FWHM is spread between 38–58 meV (see Figure 3c) at T=15 K, which seems to be approximately twice larger than for the SK QDs [37,38,40,44]. For the dots concentrated in the A family, the FWHM is several times larger (∼140 meV), similarly to the A’ band which probably has a very different origin. The PL bands broadening most likely reflects the variation in the QDs lateral dimensions, as suggested by the geometry of nanopits presented in Figure 1b. First, the pits non-uniformity is induced by the distribution of openings sizes in the silica oxycarbide mask (see discussion in Section 2.5). Second, it results from the anisotropic etching of the pits expected in III–V materials [35]. Additionally, the PL broadening may be affected by: (i) the dot-to-dot variation in chemical composition due to As to P exchange during the growth interruption under AsH${}_{3}$, prior and after the In droplet deposition, followed by the InP layer capping and temperature annealing; (ii) the dot-to-dot strain variation, as InAs and InP materials have roughly 3.2% lattice mismatch.

The analysis as mentioned above leads to speculation about the QDs height variation and the formation of multimodal QDs distribution. The size of an In droplet accumulated in a nanopit is proportional to the surface area surrounding the droplet since the semiconductor surface catalyzes the decomposition of TMIn molecules. Thus, the distribution of nanopits results in QDs size variation. It can be translated onto the QD height distribution, resulting from a difference of an integer number of InAs monolayers between adjacent QDs families, as presented schematically in Figure 1c.

### 2.3. Temperature-Dependent PL Experiment

Figure 3a shows the temperature evolution of PL emission from the examined structure. At T=300 K, there is no indication of multimodal QDs distribution. The fine structure of the PL bands appears at around 200 K while lowering the temperature. It evolves into pronounced emission modes labeled as A’ to G. The Gaussian-line shapes are fitted to the modes to extract an individual PL mode energy position, its FWHM parameter, and the mode intensity. The extracted parameters are plotted in Figure 3b–d, respectively. The fitting procedure with a multi-Gaussian function acts reasonably well up to ∼250 K, producing reliable results. The G distribution is excluded from the analysis due to a relatively low confidence level of the obtained results.

The analysis starts from Figure 3b that contains temperature evolution of the energy position of individual PL modes (symbols), and the calculated temperature-driven bandgap shift of the QD material, namely InAs (solid black lines), which is calculated according to the semi-empirical Varshni formula [46]:

(1) $$E_{\mathrm{InAs}}\left(T\right)=E_{\mathrm{InAs}}\left(T=0\right)-\left(\alpha T^{2}\right)/\left(T+\beta \right),$$

with $\alpha =2.76\;\times \;10^{-4}\;e\;V/\;K^{2}$, and β=93 K [47]. The calculated curves are shifted in energy and overlapped with the experimental data points for better comparison. The temperature shift of PL modes A–F follow the bandgap evolution of QD material quite nicely except the mode A’. This observation is in contrast to the previously observed temperature-driven PL modes evolution in multimodal-distributed SK InAs/InP QDs, where the PL band energy deviated from the predicted bandgap shift [1]. The deviation, particularly at elevated temperatures, is mainly governed by a carrier redistribution process between different families of dots and among QDs of an individual family, shifting the mean energy of related PL mode either upward (due to the filling of large dots within the distribution), or downward (due to the carrier escape from higher to lower energy dots) [48].

In the case of SK InAs/InP QDs, the carrier transfer can happen through either: (i) lateral coupling, namely carrier tunneling between QD excited states in their dense ensemble [49,50]; (ii) hybrid QD-WL states [51,52]; (iii) indirect transitions between the WL and QD energy levels [53,54]; (iv) carrier re-excitation to the WL or barrier material due to the rising phonon bath, and subsequent redistribution among QDs with higher confinement energy [1]. Accordingly, the lack of the PL mode energy deviation for the studied QDs concentrated in A–F distributions suggests the absence of direct coupling between the dots and an inefficient mutual carrier exchange which could be mediated only through the barrier states due to the absence of a WL. This property is highly beneficial for QD-based devices as thermal processes deteriorate their performance. The energy deviation from the temperature-driven InAs bandgap evolution for the PL mode A’ (shown in Figure 3b) is exactly an example of the impact of carrier re-excitation from the confining potential, in this case, most likely to the barrier states. The states represented by the PL band A’ have relatively low confinement energy since the distance of the PL mode energy (∼1.23 eV) to the InP barrier (∼1.38 eV) at T=15 K is roughly ∼150 meV. While considering the band offset distribution between strained conduction and valence bands (~40% and ~60%) the maximal confinement energy depth can be as small as ∼60 meV in the conduction band and ∼90 meV in the valence band. Even though the smallest of the estimated confinement energies can be smaller than these values, its current value roughly matches the obtained PL intensity quench energy for the band A’ displayed in Figure 3e. It leads to the conclusion that for the confined states in the A’ distribution the electron escape to the InP barrier can be the main PL intensity quench mechanism. We assume that due to very different observations regarding the PL band A’ energy distance to the adjacent A band and a shallow confinement energy resulting in the efficient PL intensity quench already at low temperatures, the A’ distribution can be described as 0D-like localized states in a thin InAsP QW of nonuniform thickness above the QDs (see Figure 1c).

The lack of carrier exchange between investigated QDs are confirmed by the analysis of the FWHM parameter displayed in Figure 3c. For the PL bands B to F (see Gaussian shapes in Figure 2b), the FWHM at T=15 K is between 38 meV and 58 meV. However, with increasing temperature, the FWHM increases to the range of 55–68 meV at T=260 K. This behavior is related to the carrier-phonon-induced broadening of an optical transition [55] without a strong signature of the carrier redistribution process, which typically results in a complicated functional form of the FWHM(*T*) [53,54,56]. The indication of the carrier re-excitation process influencing the FWHM is possibly observed for the PL band A. First, the band is significantly broadened (FWHM≈140 meV) already at T=15 K, similarly to the PL band A’ (see Gaussian shapes in Figure 2b). Then, the FWHM decreases slightly with *T* to about 120 meV at T=240 K. Since the QDs distribution A is extensive and its high energy tail overlaps with the A’ distribution of 0D-like localized states in the InAsP QW, it is probable that carriers confined in the high energy tail of the A distribution can be re-excited to the barrier states which results in slightly reduced FWHM.

The discussion is now shifted towards the temperature-induced PL intensity quench for PL bands A’ to F presented in Figure 3d. The PL intensity quench for QDs distributions A to F is quite similar. However, it is very different from that obtained for the A’ distribution. A standard PL intensity quench formula with two activation processes [57]:

(2) $$I\left(T\right)=\frac{I_{0}}{1+B_{1}\exp\left(-E_{1}/k_{B}T\right)+B_{2}\exp\left(-E_{2}/k_{B}T\right)}$$

is fitted to the experimental data points. Here, $I_{0}$ is the PL intensity at T≈0, $B_{1}$ and $B_{2}$ are relative PL intensity quench rates for the two assumed processes, and $E_{1}$ and $E_{2}$ are the characteristic activation energies. The fitting curves are displayed as solid red lines in Figure 3d while the extracted activation energies are summarized in Figure 3e. The smaller ($E_{1}$) and larger ($E_{2}$) activation energies for QDs in distributions A to F do not follow any evident trend staying at a very similar level: $E_{1}\approx 10\;me\;V$ and $E_{2}\approx 70\;me\;V$. Moreover, the activation energies are comparable to those already obtained for the previously studied ensemble of selective-area growth DE InAs/InP QDs ($E_{a,1}\approx 7\;me\;V$ and $E_{a,2}\approx 55\;me\;V$) which have been attributed to thermal processes occurring among the InP barrier states and not QDs themselves [29]. As expected, following the above-mentioned data analysis, the A’ distribution has a much lower $E_{2}$ activation energy of about 35 meV.

The results related to QDs concentrated in distributions A to F have yielded several findings. First, the qualitative similarities and the lack of mutual correlations between the PL intensity quenches strongly suggest the lack of carrier exchange between the dots, as opposed to typical observations for the matrices of multimodal SK InAs/InP QDs [1,57]. Second, quantitative similarities among the values of activation energies may point to the same PL quench mechanism independent of the dot size, which is opposite to SK InAs/InP QDs where the PL intensity quench energy scales with the QD potential depth [1]. Third, the activation energies are comparable to those previously obtained for the ensemble of SAG InAs/InP QDs [29]. They are attributed to carrier quench mechanisms in the InP barrier which seems to be also active for the studied dots.

### 2.4. Time-Resolved PL Experiment

**Figure 4:**
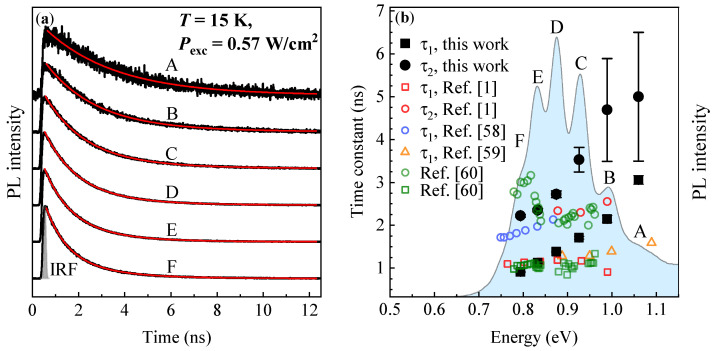
(**a**) Low-temperature (T=15 K) time-resolved photoluminescence traces for site-selective DE InAs/InP QDs measured for QD distributions A to F (black solid lines). The structure is non-resonantly excited at ∼1.55 eV with the laser power $P_{\mathrm{exc}}=0.57\;W/cm^{2}$. Red solid lines—double-exponential fit. Gray line shows the IRF of the setup with the FWHM = 80 ps. (**b**) Extracted decay time constants—$\tau_{1}$ (solid black rectangles) and $\tau_{2}$ (solid black circles) for multimodal DE QDs and their comparison to those obtained for multimodal SK InAs/InP QDs (open red, violet and orange symbols, Refs. [1,58,59]), and an ensemble of InAs/AlGaInAs/InP elongated QDs (open olive squares and circles, Ref. [60]). The blue shaded area is the PL spectrum from site-selective DE QDs.

Low-temperature TRPL traces registered for PL modes A to F of the investigated QDs are presented in Figure 4a (solid black lines). Each TRPL trace is best fitted with a double-exponential decay function, displayed as a red solid line in Figure 4a, to extract decay time constants: $\tau_{1}$ and $\tau_{2}$. We use the function of the form:

(3) $$I\left(t\right)=\left[A_{1}\exp\left(-\frac{t}{\tau_{1}}\right)+A_{2}\exp\left(-\frac{t}{\tau_{2}}\right)\right],$$

where I(t) is the TRPL intensity at time *t*, and $A_{1}$, $A_{2}$ are the amplitudes. The decay time constants are plotted as a function of the PL mode mean energy in Figure 4b (solid black circles and rectangles). The $\tau_{1}\left(E\right)$ and $\tau_{2}\left(E\right)$ trends are very suggestive, and if not generated by the increasing impact of non-radiative processes with increasing the dot size, they may provide information on the e-h pair confinement conditions.

In the early theoretical work on electron-hole dynamics in InAs/InP QDs, a similar trend was found (presented in Figure 4b with open blue circles) [58]. The decreasing e-h lifetime with increasing the dot size, as opposed to typical InAs/GaAs QDs, was attributed to increasing the overlap between electron and hole wavefunctions while expanding the dot due to the increasing confinement of carriers within the dot material boundaries [58]. The numerically estimated e-h lifetime drops from ∼2.1 ns to ∼1.7 ns in the e–h transition energy range roughly from 0.87 eV to 0.75 eV [58]. However, the simulated dots were smaller than 3 nm in height and 20 nm to 25 nm in the lateral dimensions. Previous experimental results on SK multimodal-distributed InAs/InP QDs have shown similar qualitative results [59]. However, the theory in Ref. [58] seems to overestimate the lifetime value since in the experiment the lifetime drops from ∼1.6 ns to ∼1.3 ns within the spectral range of about 1.09 eV to 0.89 eV (orange open triangles in Figure 4b) [59]. The studied dots were ∼1.5 nm in height and 30–50 nm in lateral dimensions. A possible explanation for this mismatch has been provided later [60] and it points to a strong influence of e-h Coulomb correlations significantly increasing the transition oscillator strength and thus decreasing the observed e-h lifetime. This effect is a consequence of a dense ladder of confined states observed in self-assembled SK InAs/InP QDs which have typically large in-plain sizes. The influence of Coulomb correlations on the transition oscillator strength has been recently confirmed for multimodal SK InAs/InP QDs (H∈ 0.3–2.4 nm, and 45–85 nm in-plain size) [1]. The registered PL lifetime decreased from ∼1.2 ns to ∼1.1 ns in the energy emission range of roughly 0.88 eV to 0.76 eV, completing the picture with the experimental data on multimodal SK InAs/InP QDs across the literature.

On this background, one can deduce that the $\tau_{1}$ time can represent the lifetime of e-h pairs at their ground states of the examined DE QDs. However, the significant QD size distribution, reflected in the FWHM parameter, can sweep the e-h confinement regime in a broad range from an intermediate (for large dots) to strong (for smaller dots) and even possibly a weak confinement regime (when an e-h pair is weakly bound to the QD confining potential), leading to a very pronounced trend of $\tau_{1}\left(E\right)$. At low emission energy, the $\tau_{1}$ time qualitatively and quantitatively fits to the trend for SK InAs/InP QDs for which the e-h confinement is in the intermediate regime. For higher emission energies (when the dots are getting smaller), the confinement regime tends to be weak. A particle tends to be pushed out of the QD confining potential, leading to the decrease of the e-h wavefunction overlap (and the oscillator strength) and so to the increase of the PL lifetime.

The presence of the second long-lasting decay component in TRPL traces is more puzzling. It has been shown that for InAs/AlGaInAs/InP QDs with asymmetric carrier confining potential the ground state emission at low temperature can occur via two exciton eigenstates with different oscillator strength [60,61]. It can lead to the observation of two PL decay components, displayed in Figure 4b (olive open circles and squares). For the studied DE QDs, the possible sources of the confining potential asymmetry could be, e. g., anisotropic etching of InP, strain relaxation of InAs material in the pits, and As/P material exchange. These would induce the splitting of exciton eigenstates in the QDs, leading to the observation of both short and long decay components in TRPL. Another explanation could be that the $\tau_{2}$ time can be generated by the coupling of an electron and/or a hole to the states in the surrounding InP barrier or at the InAs/InP interface. With shifting an electron or a hole wavefunction towards the dot material boundaries, one could expect the reduction of the wavefunction overlap between confined particles, elongating the e-h lifetime. Yet, this issue remains unclear and needs further investigations.

**Figure 5:**
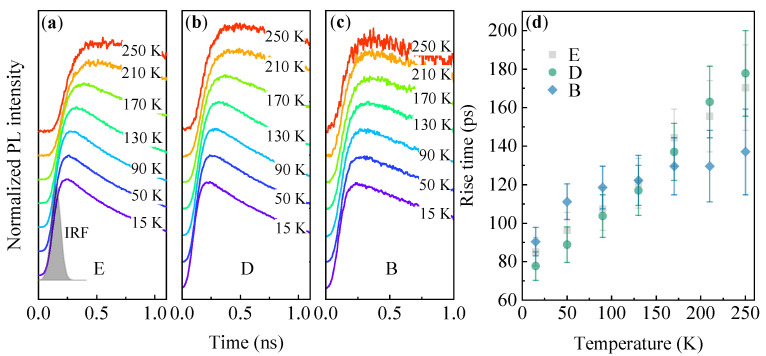
Examples of TRPL traces with highlighted temperature evolution of the PL intensity rise for the PL modes E (**a**), D (**b**), and B (**c**) for site-selective DE InAs/InP QDs. (**d**) The extracted PL rise time as a function of temperature.

An interesting feature of the studied QDs is revealed by the temperature evolution of the PL intensity rise in TRPL traces. Figure 5a–c shows exemplary traces for the PL modes E, D, and B, respectively. It is clear that with increasing the temperature, building up of the equilibrium maximal PL intensity is delayed in respect to the moment of excitation. The extracted PL rise times are collected in Figure 5d, showing monotonic increase from ∼80 ps at T=15 K, to beyond 130 ps at T=250 K. A similar observation has been recently made for an ensemble of SAG InAs/InP QDs [29], and we believe that these statements apply to the investigated dots as well. The PL rise time is contributed by two independent times: the carrier capture time, and intradot relaxation time. For typical SK InAs/InP QDs the latter is settled within the range of 1–40 ps [62,63,64,65], and the relaxation process should speed up with temperature due to increasing carrier-phonon scattering probability. Therefore, the PL rise time seems to be dominated by the capture time. It has been shown that for SK InAs/GaAs QDs when the carrier reservoir (WL) is composed of the significant density of long-lived states, the carrier capture to the dots can be strongly affected by the carrier migration process in the reservoir before the feeding of the dot states [14]. In this case, the PL rise time can significantly increase with the temperature. Confirmation of the significant density of states in the barrier seems to be seen in the temperature quench of PL intensity. Since the PL intensity quench energies are similar for all the PL modes, the quenching process occurs not in the QDs but in the barrier. The remnants of the carrier population in the reservoir are subsequently acquired by the dots.

### 2.5. Calculations of the QD Electronic Structure

**Figure 6:**
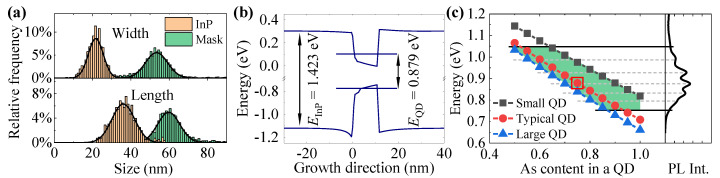
(**a**) Histograms of in-plane sizes of mask openings and nanopits etched in InP, fitted with normal distributions with standard deviation σ. (**b**) Band structure and energy levels for a typical QD geometry and composition of C${}_{\mathrm{As}}=0.75$ [marked with red square in panel (**c**)] which calculated transition energy is in the band D of the PL spectrum. (**c**) QDs transition energy (symbols) for different contents of As in an InAsP QD and for different sizes: typical QDs have sizes equal to expected values (μ) for distributions shown in (**a**), and small (large) QDs have the in-plane sizes of μ−σ (μ+σ). Height of a QD is set to half of its width. The PL signal shown on the right side helps in determination of the range of sizes and QD compositions typically realized in the structure (marked as the green area).

To support our PL data interpretation, we model the energy structure of QDs in the 8-band ***k***·***p*** approach. We assume that QDs nucleate in nanopits in the InP substrate. Therefore, the structural data presented in Figure 1a,b is used to recover the most likely realized QD geometry. Figure 6a shows the size statistics of openings in the oxycarbide mask and of the InP nanopits.

We find that the initially almost cylindrical openings in the oxycarbide mask (aspect ratio of 1.12) with average axes of $d_{1}=\left(53\pm 12\right)\;\;nm$ and $d_{2}=\left(60\pm 12\right)\;\;nm$ turn into InP nanopits of more asymmetric shape (aspect ratio of 1.67), due to the anisotropic etching of the crystallographic InP planes. The average width of the roughly rectangular InP surface openings is W=(21.6±7.8) nm and the length L=(36±12) nm. The average values estimate the expected values (μ) and uncertainties—the standard deviations (σ) of the normal distributions, fitted to the histograms and shown with solid lines in Figure 6a. The depth of nanopits cannot be recovered from the SEM images. However, the nanopit etching process is realized perpendicular to the {111}A and {111}B InP planes. Thus, the cross-section of an etched nanopit is triangular with the right angle at the apex, as schematically shown in Figure 1c. Therefore, the height of a nanopit (*H*) can be linked to its width. The established relation is H=W/2, which implies the average height of a dot to be H≈11 nm. We also determine the density of InP nanopits to be $1.55\;\times \;10^{10}\;cm^{-2}$.

From QD growth conditions, we estimate that the amount of InAs material deposited during the growth corresponds to an 0.7 nm-thick layer. Then, taking into account the nanopits surface density, we estimate the volume of QDs that acquire the InAs material: the deposited amount of InAs is enough to fill all the nanopits, and the rest of InAs material forms a thin, 0.3 nm-thick layer on the InP surface. This layer is most likely responsible for the observed PL emission of the A’ band. These estimations lead to the conclusion that the in-plain geometry of a nanopit should directly correspond to the in-plain geometry of a QD. We define a “typical” QD geometry as corresponding to the average QD sizes (μ), and “small” and “large” geometries with sizes equal to μ−σ and μ+σ of respective distributions.

For the QD band structure calculations we take into account the inter-diffusion of phosphorus atoms into the InAs QDs during the InP capping and subsequent annealing of the structure. Generally, the presence of P inter-diffusion depends on the growth conditions and the observed multimodal emission patterns have been explained either as InAsP QDs with the P intermixing on the level of 12% [43] or as pure InAs QDs [40,41]. In Ref. [44], the observed multimodal PL pattern has been explained by tight-binding calculations, and the best agreement has been found for the InAsP QDs with constant P concentration on the order of 6–10%, the exact value dependent on the growth conditions. The formation of QD families *per se* does not depend on the mere presence of the As-P exchange (resulting in the dot-to-dot variations in the chemical composition).

Therefore, the QD band structure calculations are performed in the space of two parameters: QD geometry and chemical composition. Figure 6b shows the band structure for the “typical” QD, plotted along its growth direction, together with calculated fundamental electron and hole energy levels. The ground state energy of the dot is defined as the difference between these states. The geometry of the QD is W=22 nm, L=36 nm, H=11 nm, and the As content in the QD is C${}_{\mathrm{As}}=0.75$, marked also with the red square in Figure 6c. Note that the energy differences between the QD electron fundamental level and the conduction band edge in InP (199 meV), and between the hole fundamental level and the valence band edge in InP (345 meV) are much higher than the observed PL intensity quench energies. This further supports our interpretation of the temperature-dependent PL.

After the calculations of single-particle electron and hole states in the QD confining potential as a function of the As content in an InAsP QD, the transition energies are plotted in Figure 6c and compared with the low-temperature PL spectrum (right side of the panel). These results allow us to estimate the region of potential QD chemical composition that can result in the observed PL energies of QDs (for the most frequent QD sizes). This region with boundaries set by the small and large QD geometries is shown with green area in Figure 6c. The low energy side of the spectrum (peak G at 0.752 eV) can be explained by the large dots with C${}_{\mathrm{As}}\approx 0.85$, or pure InAs QDs smaller than the typical size. Similarly, the high energy side of the spectrum connected with the QD emission (peak A at 1.049 eV) can originate from large dots with C${}_{\mathrm{As}}\approx 0.50$, or small dots with C${}_{\mathrm{As}}\approx 0.65$. Note that all the intermediate geometries and compositions between those mentioned may be realized in the investigated QDs. In result, the determined space of QD size and composition should be interpreted as a calculation-based proof that the observed multimodal PL pattern comes from the QD emission, without giving a definite explanation of QD geometries and compositions that contribute to the PL spectrum.

## 3. Experimental and Theoretical Methods

### 3.1. Fabrication of Nanopits

We use the low-pressure (80 mbar) MOVPE TurboDisc^®^ D125 reactor (EMCORE) with TMIn, tertiarybutylphosphine (TBP), AsH${}_{3}$ and phosphine (PH${}_{3}$) as precursors, and hydrogen (H${}_{2}$) as a carrier gas. The mask fabrication is described in Ref. [29] and starts with the spin-casting the PS-*b*-PDMS powder (61–111 K, purchased from Sigma-Aldrich, St. Louis, MO, USA) dissolved in cyclohexane 1/400 mg/mL onto the InP(100) oriented wafer with a predeposited InP epitaxial buffer layer (500 nm of InP grown with $6.24\;\times \;10^{-2}\;m\mathrm{mol}/\min$ flow of TMIn and 8.9 mmol/min of PH${}_{3}$). The solvent annealing (methylcyclohexane saturated vapor) promotes the formation of the pattern of standing PS cylinders in the PDMS matrix. This structure is transformed into the hard silicon oxycarbide mask in the inductively coupled plasma-reactive ion etching (ICP-RIE) process where the oxygen plasma oxidized the PDMS matrix and removed the PS cylinders leaving the hexagonal pattern of openings with sizes as shown in Figure 6a. Afterwards, the wafer is cleaned with concentrated H${}_{2}$SO${}_{4}$ and loaded into the MOVPE reactor, where the nanopits are etched in the InP substrate using CBr${}_{4}$ for 100 s at $550\;{}^{\circ }C$ ($3.2\;\times \;10^{-4}\;m\mathrm{mol}/\min$) under the phosphine ambient (PH${}_{3}$ flow of 8.9 mmol/min with TBP flow of 3.2 mmol/min), and subsequently the mask is removed by hydrofluoric acid (HF). The SEM images have been registered with the Zeiss Supra SEM 40VP in-lens detector, and the electron acceleration voltage of 2 KV and magnification of 50 kX for Figure 1a and 133.82 kX for Figure 1b. The brightness is set to 47% and contrast to 28%.

### 3.2. Droplet Epitaxy of QDs

The QDs nucleation is directly preceded by InP wafer annealing at $550\;{}^{\circ }C$ for 15 min in the MOVPE chamber under phosphine ambient (8.9 mmol/min flow of PH${}_{3}$ and 1.8 mmol/min flow of TBP), then ramping the temperature to $480\;{}^{\circ }C$ with the PH${}_{3}$ and TBP fluxes maintained, and the growth interruption for 30 s under $5.52\;\times \;10^{-2}\;m\mathrm{mol}/\min$ of AsH${}_{3}$ flow. The indium droplets are deposited in the openings (in the absence of arsine in the growth chamber) at $480\;{}^{\circ }C$ (TMIn flow of $2.81\;\times \;10^{-2}\;m\mathrm{mol}/\min$ for 5 s), and subsequently annealed for 60 s under $5.52\;\times \;10^{-2}\;m\mathrm{mol}/\min$ AsH${}_{3}$ flow, promoting their crystallization into InAs QDs, following the InP crystal lattice. The QDs layer is capped with nominally 1 nm-thick InP layer at $480\;{}^{\circ }C$ (with vapor flows of TMIn $2.81\;\times \;10^{-2}\;m\mathrm{mol}/\min$, PH${}_{3}$ of 8.9 mmol/min, and TBP of 1.8 mmol/min), and annealed for 120 s in phosphine ambient with maintained flows of PH${}_{3}$ and TBP, while the temperature is rising up to $570\;{}^{\circ }C$ in order to facilitate the out-diffusion of point defects. Finally, the 2 nm-thick InP layer is deposited at $570\;{}^{\circ }C$ with the vapor flows identical to the 1 nm-thick InP layer.

### 3.3. Optical Methods

For the optical characterization, the structure with site-selective DE InAs/InP QDs is held in a cryostat allowing for the control of temperature in the range of 15–300 K. For the excitation power density- and temperature-dependent experiments, the structure is excited non-resonantly with a continuous-wave semiconductor laser diode (neodymium-doped yttrium aluminum garnet, λ=532 nm). The mode-locked Ti:Sapphire laser is used to produce trains of ∼2 ps long pulses at the frequency of 76 MHz, which excites the structure non-resonantly (λ=800 nm) for the TRPL studies. The far-field optical setup is used to collect the PL signal, which is spectrally resolved by a 0.3 m-focal-length monochromator. The time-integrated PL spectra are measured via the lock-in technique, using an InGaAs photoconductive single-channel detector (Hamamatsu Photonics, Hamamatsu City, Japan). TRPL is measured via time-correlated single-photon counting exploiting an NbN superconducting single-photon counting detector with 80–90% efficiency and 200 dark counts per second at telecom range and a multichannel picosecond event timer as the time-to-amplitude converter. The overall time-resolution of the setup is ∼80 ps.

### 3.4. Calculations of the QD Band Structure

Numerical calculations of the QDs band structure are performed with the commercially available *nextnano* software (nextnano GmbH, Poing, Germany) [66], which uses the continuum elasticity model for the determination of the strain distribution, and afterwards calculates the eigenstates using the 8-band ***k***·***p*** method, including the strain-driven piezoelectric field. Relevant material parameters are taken from Ref. [47]. This approach does not take into account the Coulomb interaction; however, it is sufficient for the determination of the band structure and the energy levels.

## 4. Conclusions

In conclusion, we present the optical properties of InAs/InP QDs fabricated site-selectively by the droplet epitaxy technique. The predefined positioning of the QDs is provided by block copolymer lithography leading to fabrication of densely distributed nanopits in the InP substrate acting as QD nucleation sites. The upper constrain of the QDs density is $∼1.55\;\times \;10^{-10}\;cm^{-2}$, based on the SEM image analysis of the InP surface with nanoholes. Additionally, the statistical analysis provides the average in-plain QD dimensions, which are (36±12)nm in length and (21.6±7.8)nm in width, with the resultant in-plain aspect ratio of 1.67. We estimate the average QD height as ∼11 nm, taking into account the right angle at the nanopit apex, defined by the anisotropy of the wet etching.

Room-temperature PL experiment presents an intensive emission band centered at 1.5 μm. The PL shows considerable inhomogeneous spectral broadening attributed to the substantial dot-to-dot variation in size and in chemical content within the QDs ensemble. At T>250 K, the emission spectrum reveals a fine structure consisting of a few PL bands with the non-linear band-to-band energy separation suggesting that the dots are multimodal-distributed. This distribution can be related to both non-uniform etching rates for nanopits and an inhomogeneous distribution of indium droplets.

The low-temperature excitation power-dependent PL experiment shows that the PL response from the dots in contributed by the ground state emission over three decades of the excitation power density, without signatures of the higher-energy states emission process. It seems to be consistent with the QD band structure calculations, showing mostly a single electron state confined in a dot.

Temperature-dependent PL experiment determines that: (a) the PL bands follow expectedly well temperature-driven bandgap evolution of InAs material; (b) the spectral broadening only slightly increases with temperature; (c) the PL intensity quench remains qualitatively and quantitatively similar for all the QD families. This suggests a relatively good electron-hole confinement in the dots and the lack of carrier transfer between QDs with temperature, the latter being very different from the SK InAs/InP QDs. Secondly, this observation points towards the existence of trap states in the InP barrier providing efficient non-radiative carrier recombination centers causing the carriers to relax the energy before they are captured by QDs. The time-resolved PL experiments underline the role of the barrier states revealing an increase in the PL rise time from ∼80 ps to ∼130 ps with increasing temperature. The elongation of the PL rise time is assigned to the prolonged carrier transfer within InP barrier states before electrons and holes are trapped by QDs.

The multimodal character of QDs emission allows for the investigations of the confinement strength for electron-hole pairs in QDs, reflected in the electron-hole recombination process. The time-resolved PL experiment shows two decay components strongly dependent on the QD emission energy. The fast component changes between $\tau_{1}\approx 0.9\;ns$ and $\tau_{1}\approx 3.1\;ns$ when shifting from large dots of the lower emission energy to smaller dots with higher emission energy. This confirms a broad size distribution of the dots, revealing that confinement regime is scattered from weak to strong. A similar spectral characteristic is found for the second, long-lasting PL decay component dispersed from $\tau_{2}\approx 2.2\;ns$ at ∼0.8 eV energy emission to $\tau_{2}\approx 5.0\;ns$ at ∼1.07 eV. However, the origin of the $\tau_{2}$ component remains unclear.

Finally, the numerical calculations of the QD band structure confirm our experimental findings, underlying the role of As-P intermixing in the QD material. The single-particle ground state energies and transition energies, calculated for the average QD geometry (provided by the analysis of the nanopits pattern and the etching procedure), show that the dots can be indeed realized with the assumed geometries. The calculated transition energies fit well to the measured PL spectrum. However, the space of considered parameters must involve chemical variation in the QD material, suggesting the As-P intermixing in the range of 0–50%.

Presented research shows comprehensive studies on site-selectively grown InAs/InP QDs with predefined positioning by block copolymer lithography. The dots show good optical response with emission wavelength centered at the application-relevant telecom range. However, sizeable inhomogeneous broadening of the QDs ensemble and the detrimental role of charge traps and non-radiative centers in the barrier must be addressed. We believe that the optimization of the mask preparation process, chemical etching conditions, InP surface treatment, and InAs (QD) deposition can increase the overall quality of the dots at the level acceptable for device applications.

## Data Availability

The data that support the findings of this research are available from the corresponding author (P. H.) upon reasonable request.

## Abbreviations

The following abbreviations are used in this manuscript:

DE	Droplet epitaxy
e-h	Electron-hole
FWHM	Full-Width-at-Half-Maximum
ICP-RIE	Inductively coupled plasma-reactive ion etching
MOVPE	Metalorganic vapor-phase epitaxy
PL	Photoluminescence
PS-*b*-PDMS	Polystyrene-block-polydimethylsiloxane
QDs	Quantum dots
QW	Quantum well
SAG	Selective-area growth
SEM	Scanning electron microscope
SK	Stranski–Krastanov
TBP	Tertiarybutylphosphine
TRPL	Time-resolved photoluminescence
TMIn	Trimethylindium
WL	Wetting layer
